# New Drugs, Appliances, and Things Medical

**Published:** 1890-10-25

**Authors:** 


### NEW DRUGS, APPLIANCES, AND THINGS MEDICAL.

### PROFESSOR NEUGEBAUER'S NEW DISINFECTANT.

We have received a specimen of the above for notice. It consists of a compressed crystalline white mass, about half an inch thick, and mounted in a neat frame. This is intended to be hung up near the ceiling of sick rooms, nurseries, and those offices most exposed to unhealthy emanations. The material is not very soluble in water, completely so in spirit, and is entirely volatile by heat. It thus fulfils the conditions for a solid volatile disinfectant and deodorizer. It has a pleasant oakum-like smell. We think it is likely to be largely employed, as the mechanics of its use are so conveniently simple—merely a nail in the wall to hang the tablet-frame on—and also because the materials employed are powerful agents for the destruction of disease-causing germs and noxious effluvia. The agents and importers are Messrs. W. H. Power and Co., 30, Budge Row, Cannon Street.

